# Stool-based biomarkers of interstitial cystitis/bladder pain syndrome

**DOI:** 10.1038/srep26083

**Published:** 2016-05-18

**Authors:** A. Braundmeier-Fleming, Nathan T. Russell, Wenbin Yang, Megan Y. Nas, Ryan E. Yaggie, Matthew Berry, Laurie Bachrach, Sarah C. Flury, Darlene S. Marko, Colleen B. Bushell, Michael E. Welge, Bryan A. White, Anthony J. Schaeffer, David J. Klumpp

**Affiliations:** 1 Carl R. Woese Institute for Genomic Biology, University of Illinois at Urbana-Champaign, 1207 West Gregory Drive, Urbana, Illinois, USA; 2 Applied Research Institute at University of Illinois at Urbana-Champaign, 1205 West Clark Street, Urbana, IL, USA; 3 Department of Urology, Feinberg School of Medicine, Northwestern University, 303 East Chicago Avenue, Chicago, IL, USA; 4 Department of Microbiology-Immunology, Feinberg School of Medicine, Northwestern University, 303 East Chicago Avenue Chicago, IL, USA

## Abstract

Interstitial cystitis/bladder pain syndrome (IC) is associated with significant morbidity, yet underlying mechanisms and diagnostic biomarkers remain unknown. Pelvic organs exhibit neural crosstalk by convergence of visceral sensory pathways, and rodent studies demonstrate distinct bacterial pain phenotypes, suggesting that the microbiome modulates pelvic pain in IC. Stool samples were obtained from female IC patients and healthy controls, and symptom severity was determined by questionnaire. Operational taxonomic units (OTUs) were identified by16S rDNA sequence analysis. Machine learning by Extended Random Forest (ERF) identified OTUs associated with symptom scores. Quantitative PCR of stool DNA with species-specific primer pairs demonstrated significantly reduced levels of *E. sinensis*, *C. aerofaciens*, *F. prausnitzii*, *O. splanchnicus*, and *L. longoviformis* in microbiota of IC patients. These species, deficient in IC pelvic pain (DIPP), were further evaluated by Receiver-operator characteristic (ROC) analyses, and DIPP species emerged as potential IC biomarkers. Stool metabolomic studies identified glyceraldehyde as significantly elevated in IC. Metabolomic pathway analysis identified lipid pathways, consistent with predicted metagenome functionality. Together, these findings suggest that DIPP species and metabolites may serve as candidates for novel IC biomarkers in stool. Functional changes in the IC microbiome may also serve as therapeutic targets for treating chronic pelvic pain.

Patients with urologic chronic pelvic pain syndromes (UCPPS) suffer chronic pelvic pain and dramatically lower quality of life, yet diagnostic markers and effective therapies remain elusive for these costly syndromes^1^. Interstitial cystitis/bladder pain syndrome (IC/BPS or IC) is a debilitating UCPPS condition of pelvic pain and voiding dysfunction that afflicts as many as 8 million U.S. women where depression is a common co-morbidity^2^,^3^,^4^,^5^. IC etiology remains unknown, but urothelial lesions and lamina propria mast cells are associated with patient symptoms^6^,^7^,^8^. HPA axis dysfunction has been implicated in female and male patients and cats with feline IC^9^,^10^,^11^,^12^,^13^, and thus may be common among UCPPS, but mechanisms that integrate pelvic pain, voiding dysfunction, HPA activity, and depression are lacking. Because of these long-standing questions, NIDDK has launched its flagship urology study, the Multi-Disciplinary Approaches to Chronic Pelvic Pain (MAPP) Research Network with the most comprehensive studies to date of UCPPS including clinical characterization and epidemiologic and mechanistic studies^14^,^15^.

The microbiome is increasingly appreciated as driving diverse physiologic processes in both health and disease^16^,^17^. With the notable exception of *C. difficile* colitis, early studies implicated pathogenic dysbioses primarily at the phylum and genus levels, but more recent studies identify individual species associated with disease. Moreover, individual species within the microbiome have recently been associated with driving disease through altered innate metabolism or altered pharmaceutical metabolism^18^. Despite these advances, it remains unclear whether microbiota contribute to pain syndromes generally and to UCPPS in particular. Consistent with the recent finding of a normal female urinary microbiome^19^, the MAPP Network is also exploring UCPPS microbiota with a focus on the urinary microbiome. MAPP Network studies have identified an association between fungi and symptom flares, but a signature UCPPS urinary microbiome has not yet been found that is associated with baseline symptoms. As a result, there is currently no understanding of the UCPPS gut microbiome that may modulate urinary sensation.

We recently defined bacterial pain phenotypes in murine UTI, where transient bladder infection can elicit a spectrum of pain responses, including chronic pelvic pain^20^,^21^. Because pelvic pain is subject to modulation via organ crosstalk^22^,^23^,^24^,^25^,^26^, we hypothesized that GI and/or reproductive tract microbiota modulate IC pelvic pain. Here, we characterized microbiota of IC patients and healthy controls, including MAPP participants. We observed significant differences between microbiota of patients and controls, including specific species, thereby potentially identifying novel biomarkers and potential therapeutic targets for IC.

## Results

### Clinical samples obtained from IC patients and controls

Female healthy controls and patients were recruited from MAPP participants at Northwestern and through separate advertising to yield a cohort of average age of 35 years (see Table 1). Individuals receiving antibiotic therapy within the prior 3 months were excluded from the study. Study participants exhibited racial skewing such that all IC patients were Caucasian, and three IC patients were on disability due to IC. Patients had a mean duration of symptoms of 9 years and exhibited significantly higher combined GUPI scores and significantly higher GUPI subscores. Patients also reported higher incidence of conditions previously associated with IC including allergies/respiratory tract disorders and gastrointestinal disease as well as urologic/gynecologic surgeries that represent a potential risk factor for pelvic pain.

### IC patients have altered stool microbiome

**Figure 1:**
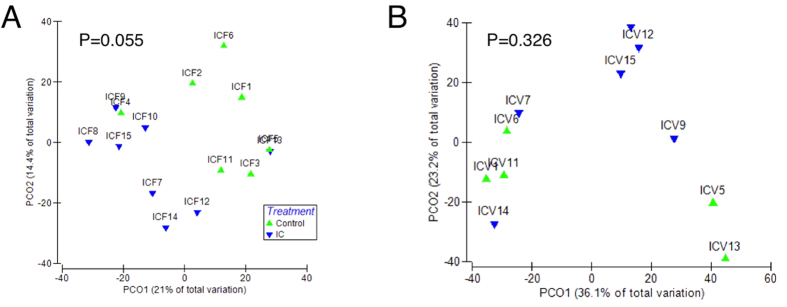
Stool and vaginal microbiomes in IC. (**A)** Principle component analysis of pilot cohort suggested segregation of stool microbiome among IC patients and controls (P = 0.055). (**B)** Principle component analyses of vaginal samples from pilot cohort did not segregate by diagnoses (P = 0.326).

**Figure 2:**
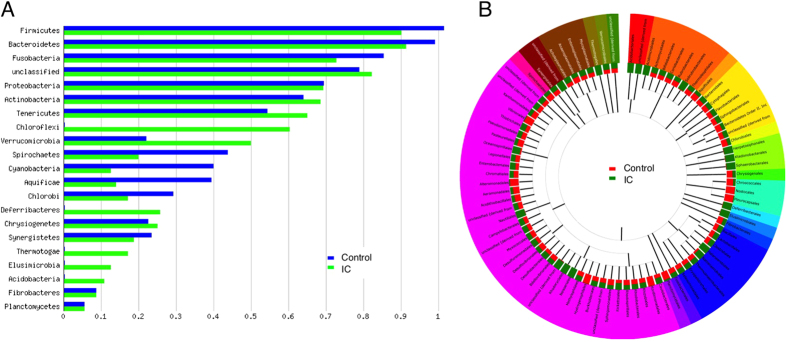
Altered microbiome in IC. **(A)** Comparison of phyla abundance between IC patients and controls as determined by 16S reads. (**B)** Comparison of bacterial order abundance shows numerous orders that are differentially abundant in controls (red) and IC (green; n = 34). Phyla are indicated by colors in the outer ring. Data were compared to M5RNA using a maximum e-value of 1e-20, a minimum identity of 97%, and a minimum alignment length of 15 bp.

DNA was purified from stool and vaginal samples, and 16S sequences of the V3–V5 hypervariable regions were amplified and subjected to deep sequencing^27^. Sequencing reads were subsequently assigned to operational taxonomic units (OTUs) using QIIME with a threshold for homology of 97%. Preliminary characterization performed on a pilot cohort revealed no trend of segregation of vaginal samples, whereas similar analyses identified stool as a potential site where microbiota of IC patients segregated from controls (Fig. 1A,B). As a result, subsequent analyses focused on the stool microbiome (Fig. 2; n = 34). Several phyla and bacterial orders were differentially represented in controls and IC patients, however these differences were restricted to less abundant phyla (Fig. 2A). For example, the three orders of Chloroflexi (Herpetosiphonales, Ktedonobacterales, and Sphaerobacterales) were virtually absent from healthy controls, yet their presence in IC represented only 0.02% of reads (Fig. 2B and data not shown). Nonetheless, these differences suggest that the IC microbiome may be functionally distinct from the normal adult fecal microbiome. Moreover, these differences at the family level suggest differences at the genera level that are associated with symptom severity.

**Figure 3:**
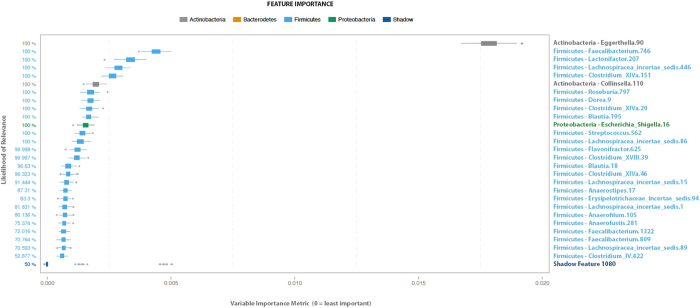
ERF identifies significant features of IC microbiome. Box plot of likelihood of relevance for fecal OTUs by (vertical) and importance (x axis; n = 34).

IC is a symptom-based diagnosis, so we sought to identify features of the IC microbiome specifically associated with patient symptoms. To identify specific taxa that discriminate IC stool microbiota from controls in association with symptom score, individual taxa were evaluated for significance as features using Extended Random Forest (ERF). Briefly, ERF uses the Random Forest (RF) algorithm followed by stability analysis and feature permutation to determine all relevant taxa. RF shows excellent performance with this type of complex data where the number of variables is orders of magnitude larger than the number of patient samples^28^. ERF analyses of stool operational taxonomic units (OTUs) revealed 26 features with a likelihood of relevance exceeding 70%. In ERF, the best performing shadow (or “noise”) feature is set at 50% and every OTU above is likely to contribute to discriminating IC microbiome from controls (Fig. 3). Significant OTUs were identified among diverse phyla including Actinobacteria, Firmicutes, and Proteobacteria. More importantly, these findings support the possibility that individual species reside within the identified taxa that are significantly altered in IC.

### Fecal species altered in IC

**Figure 4:**
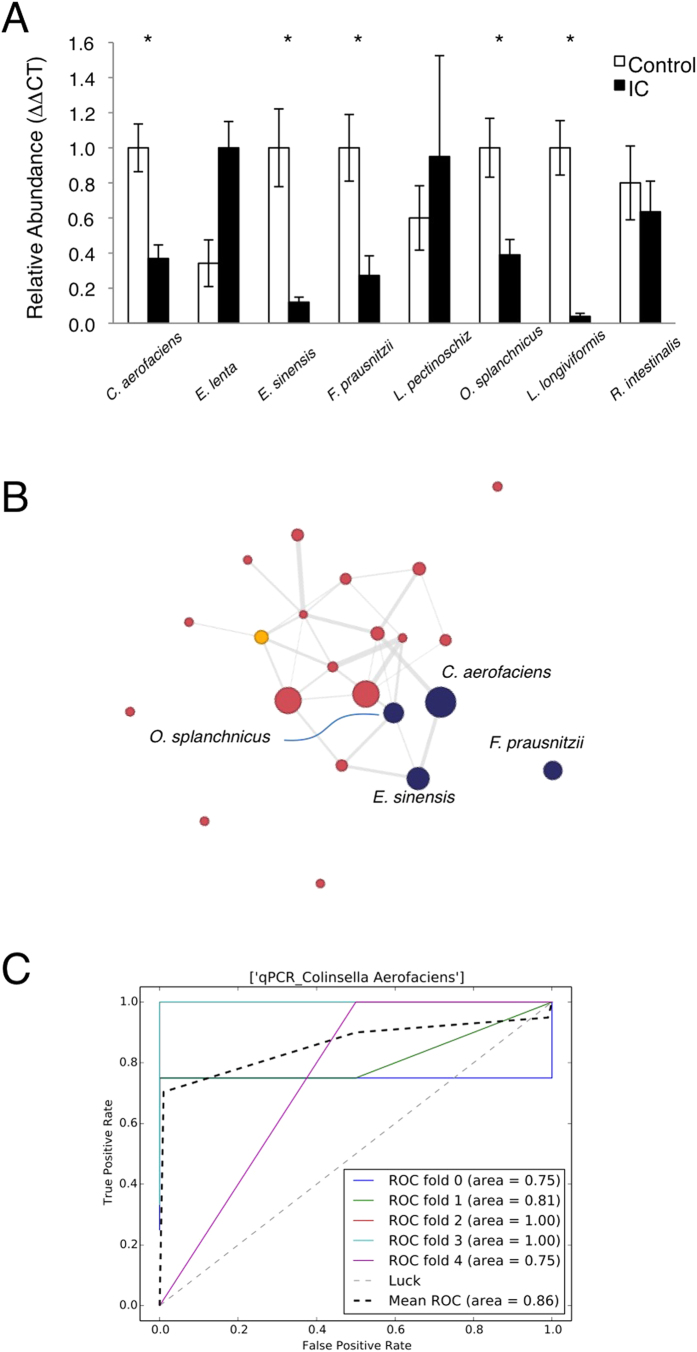
Specific stool OTUs are associated with IC. (**A)** qPCR was performed on healthy control (n = 10) and IC stool DNA (n = 16) using species-specific primers (±SEM). Species with significant, differential abundance are indicated (*P < 0.05). (**B)** Interactive network diagram integrating qPCR findings with 16S data by MIC analysis to detect relationships between features. (**C)** ROC curves for *C. aerofaciens* qPCR data.

To determine whether individual species are differentially represented in IC patient stool samples relative to controls, quantitative PCR was performed with primers designed against the top taxa ranked by ERF. DNA was purified from all stool samples of patients and controls. Species-specific primers were designed against unique regions of 16S rDNA for the top three ranked OTUs in the pilot cohort (*Odoribacter, Faecalibacterium, and Lachnospiriceae)* and the top six ranked OTUs in the ranking for the combined cohort (*Faecalibacterium, Colinsella, Eggerthella, Lachnospiriceae, Lactonifactor and Roseburia).* Because several of these OTUs were classified only at the genus level, species-specific primers were designed against all available 16S sequences in Genbank within a genus. Ultimately, specific primers were designed for *O. splanchnicus, O. denticanus, O. laneus, F. pruasnitzii, Lachnospiriceae pectinoschiz, C. aerofaciens, C. intestinalis, C. stercoris, E. lenta, E. sinensis, Lactonifactor longiviformis, and R. intestinalis.* We omitted analyses of genera for which many species were reported (e.g., *Clostridia*). Each primer pair was then tested for amplicon specificity by melt curve analysis of amplification products using a small panel of stool DNAs purified from healthy controls. Using qPCR of stool DNA from IC patients and controls, five significant species were identified: *O. splanchnicus*, *F. prausnitzii*, *C. aerofaciens*, *E. sinensis*, and *L. longoviformis* (Fig. 4A, P < 0.05). Interestingly, these species were deficient among IC patients and thus were dubbed “deficient in interstitial cystitis pelvic pain” (DIPP). Connectivity analyses were also performed by Maximal Information Coefficient (MIC) analysis to identify co-varying OTUs^29^. *C. aerofaciens*, *O. splanchnicus*, *E. sinensis*, and *F. prausnitzii* were found to co-vary, therefore suggesting that DIPP species are members of a community that is altered in IC (Fig. 4B).

### DIPP species and stool metabolites as candidate IC biomarkers

**Figure 5:**
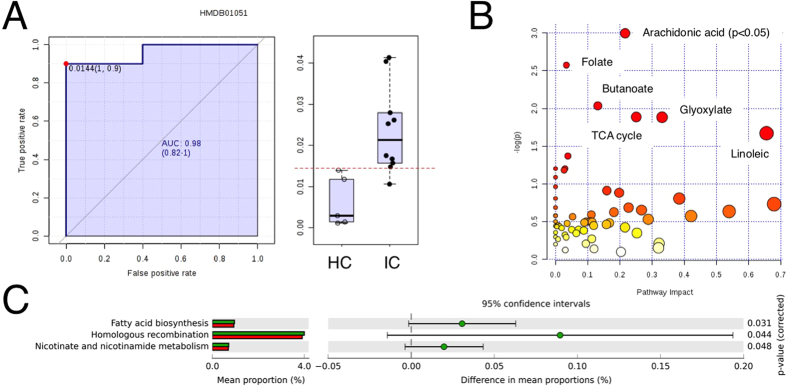
Metabolomic and metagenomic comparison in IC. (**A)** ROC analysis (left panel) and box-and-whisker plot (right panel) identified glyceraldehyde as significantly elevated in IC and a candidate biomarker (AUM = 0.98, P<0.05). (**B)** Pathway analysis of stool metabolites in MetaboAnalyst 3.0 identified arachidonic acid signaling as significantly altered in IC (P<0.05). (**C**) PICRUSt metagenome analyses identified fatty acid biosynthesis, homologous recombination and nicotinate/nicotinamide metabolism as significantly different.

To complement the microbiome analyses, we performed metabolomic studies of stool from healthy controls and IC patients by GC-MS to quantify distinct metabolites^30^. As most samples were initially stored directly in preservative, analysis was necessarily confined to a subset of samples from patients where a portion of unadulterated stool was retained (n = 15). Nonetheless, metabolomic data were subsequently analyzed for significant pathways and biomarker relevance using MetaboAnalyst 3.0^31^,^32^,^33^. Pathway analysis identified multiple candidate pathways, including lipid metabolism pathways for linoleic acid, butanoic acid, and arachidonic acid (Fig. 5A). Arachidonic acid signaling was identified as significant (P < 0.05).

Metabolites and DIPP species were evaluated as potential biomarkers for IC. Receiver operator characteristics (ROC) were determined for each metabolite by classic univariate analysis in MetaboAnalyst, where a marker was considered significant with area under the curve (AUC) in excess of 0.70. The metabolite with the best predictive value was identified as glyceraldehyde, a metabolite elevated in IC stool (Fig. 5B and Table 2; HMDB01051, AUC 0.98). Among the bacterial DIPP species, ROC analyses revealed *C. aerofaciens*, *E. sinensis*, *F. prausnitzii*, and *O. splanchnicus* as exceeding AUC > 0.70 (Table 2). Together, these findings suggest that candidate stool-based biomarkers for IC may be identified among both DIPP species and metabolites.

## Discussion

UCPPS remain diagnoses of exclusion due to a lack of biomarkers. Here, we used a combination of microbiome and metabolome analyses as a strategy to identify novel stool-based biomarkers for IC. Individual IC patients can be exquisitely sensitive to specific comestibles, where dietary constituents such as caffeine, acid, and alcohol often exacerbate pain and urinary symptoms^34^. As a result, a front-line approach to symptom management is dietary modification through an “elimination diet” to identify and then eliminate any sensitizing comestibles that vary with the individual patient^35^. We anticipated that such dietary modification among IC patients might complicate analysis of any UCPPS pathogenesis-associated shifts in stool microbiota. Thus, it was not surprising that the global level of analysis represented by PCA revealed only modest segregation of control and IC stool microbiota in the pilot cohort (Fig. 1), but these findings did not preclude identification of differences between IC and controls at the levels of phyla and order (Fig. 2), suggesting the presence of specific taxa as candidate biomarkers for IC. Indeed, more detailed analyses revealed multiple such genera.

ERF identified 26 taxa as highly significant features, including Actinobacteria, Firmicutes, and Proteobacteria (Fig. 3). An important asset of ERF as applied in these studies is that feature rankings were based on symptom scores, thus significant taxa were not identified merely on the basis of prevalence but instead reflected IC symptom severity. This is a particularly important consideration, given that UCPPS are symptom-based diagnoses that are made after excluding other conditions that might elicit similar symptoms (e.g., bladder cancer). We used the ERF findings to develop species-specific primers and validated *O. splanchnicus*, *F. prausnitzii*, *C. aerofaciens*, *E. sinensis*, and *L. longiviformis* as DIPP species with significantly lower abundance in IC stool (Fig. 4). Although all significant species were DIPP species, we speculate that the IC microbiome is not simply characterized by species deficiency but also harbors species that are increased in IC pelvic pain (IIPP). For example, albeit not significant, we note that *E. lenta* is somewhat elevated and thus is a candidate IIPP species that will be examined in future UCPPS studies (Fig. 4). Similarly, several bacterial orders were elevated in IC relative to controls (Fig. 2), suggesting that multiple genera may harbor IIPP species in the IC microbiome. As the DIPP species were initially identified on the basis of ERF association with symptom scores, it will also be interesting to determine if any or all of these species vary with individual symptom scores, such as during “flares” or periods of remission.

The IC fecal microbiome and metabolome suggest a GI contribution to UCPPS symptomatology. Little is known of *O. splanchnicus* metabolism, but *L. longoviformis* metabolizes phytoestrogens, and IC has a strong gender bias that is mimicked in a clinically relevant murine model^36^,^37^. *E. lenta* is associated with bacteremia but more recently has been implicated in digoxin metabolism, thereby shaping pharmacologic therapy^18^. Thus it is possible that *E. sinensis* also alters the IC metabolome and may therefore contribute to alterations in the gut-brain axis or pelvic organ crosstalk^22^,^38^,^39^. *F. prausnitzii* is an anaerobe that is highly abundant in the human GI tract. Interestingly, *F. prausnitzii* provides butyrate to the gut, and *F. prausnitzii* deficiency is associated with irritable bowel disease^40^,^41^,^42^,^43^. Short chain fatty acids are appreciated as beneficial mediators of the microbiome^44^,^45^, and we note that butyrate (a.k.a. butanoate) is one of the top-ranked pathways in the IC fecal metabolome (Fig. 5C). Therefore, *F. prausnitzii* deficiency may be a major mediator of the IC microbiome. Similarly, *C. aerofaciens* deficiency is significantly associated with symptoms in IBS patients^46^. Consistent with these features of the IC microbiome, PICRUSt functional prediction of the IC metagenome revealed that fatty acid biosynthesis was the most significant functionality (Fig. 5C, P = 0.03). These metagenome findings dovetail with the metabolome analyses that identified arachidonic acid, butyrate/butanoate, and linoleic acid (Fig. 5A). These findings, in turn, are supported by recent suggestions that the balance of omega-3 and omega-6 polyunsaturated fatty acids influence urologic inflammation (reviewed in^47^). Taken together, as the abundances of individual DIPP species co-vary as an apparent community (Fig. 4B), it is possible that the lipid-specific effects of any individual DIPP species on IC symptoms is compounded as the DIPP community declines and impacts multiple lipid classes that mediate urologic health and disease.

These studies have potential for important clinical implications for IC. First, IC diagnosis remains difficult for the clinician and patient alike, but stool-based biomarkers identified here offer important promise (Table 2). These candidates identified are likely not exhaustive, as these initial studies are marked by a limited cohort size, and as some genera were excluded in our analyses as a matter of convenience due to the many known species. Moreover, it is likely additional biomarker species exist beyond the DIPP species identified here because they reside within genera that did not reach the significance threshold ERF. For example, *O. splanchnicus* emerged as a candidate because ERF analyses performed on the pilot cohort initially identified *Odoribacter* as a significant OTU (this fluctuation is due to the small patient sample size of the pilot cohort). We nonetheless retained *Odoribacter* as a taxon of interest and thereby identified *O. splanchnicus* as a DIPP species. Despite these limitations, it is clear that stool is a convenient source of sensitive and specific IC biomarkers, and we identified here 6 candidate biomarkers for further study (Table 2) and others are likely to emerge. Second, and perhaps more importantly, the IC microbiome suggests probiotic/prebiotic therapeutic opportunities. For example augmenting DIPP species themselves or supplying other species with similar metabolic activities may complement metabolic defects that contribute IC directly, by the gut-brain axis, or by pelvic organ crosstalk^39^,^48^. Thus in summary, the IC microbiome and metabolome identify important new diagnostic and therapeutic opportunities for IC patients. Future studies already underway in the MAPP Network will determine if men suffering chronic prostatitis/chronic pelvic pain syndrome also exhibit an altered fecal microbiome, and it will be of great interest to determine the extent to which our IC microbiome findings can be generalized to other UCPPS conditions.

## Methods

### Study Design and Oversight

Studies were conducted under Protocol STU00055668. All experimental protocols within STU00055668 were approved by the Institutional Review Board of Northwestern University and carried out in accordance with the approved procedures, and the study was posted on ClinicalTrials.gov. Study design was unblinded, and all samples were de-identified. Participants were enrolled by a research coordinator, and all participants provided written, informed consent by consent form with valid IRB date stamp; signed consents obtained after 01 August 2014 were logged immediately into eNOTIS/NITRO. Clinical samples were collected in the Urology Clinic or by self-collection by study participants (see below). Analyses were performed at Northwestern University and under IRB exemption at University of Illinois at Urbana–Champaign.

### Patients

Female participants were recruited into the Urology Clinic of Northwestern University Feinberg School of Medicine in two separate cohorts. A pilot cohort of 15 individuals (8 healthy controls, 7 IC) was recruited from individuals participating in the NIDDK MAPP Network study at Northwestern^15^, and stool and vaginal swabs were obtained from this group. A second cohort was recruited independent of MAPP Network participation for collection of stool samples only (i.e., no vaginal swabs were collected; 9 healthy controls, 10 IC). In all cases, demographic data, medical history, and symptom scores were captured using an abbreviated MAPP demographic and medical history, and a female-specific MAPP genitourinary pain index (GUPI) questionnaire at the time of enrollment^49^. Individuals attesting to antibiotic use within the prior three months were excluded.

### Sample collection and preparation

Clinical samples of vaginal swabs and/or stool were collected from IC patients or controls at Northwestern according to protocols of the HMP Manual of Procedures. Briefly, participants were provided with a stool collection kit for at-home sample collection. Participants were instructed to freeze samples overnight and then ship to the laboratory within 48h on wet ice. Upon receipt in the laboratory, stool was aliquoted, placed into RNAlater (MoBio) and stored at −80°. For vaginal swabs, pH was determined with pH strips, and swabs were collected from the introitus, midpoint, and fornynx. Swabs were placed immediately into RNAlater and stored at −80°. Collected samples were shipped on dry ice to University of Illinois Urbana–Champaign for analyses.

### Microbiome analyses

Phylotype profiles of the microbiome populations were generated using deep amplicon sequencing of the hypervariable V3–V5 region of the 16S ribosomal RNA (rRNA) gene, which has been validated by the HMP for use with human microbiomes^27^. Barcoding samples prior to MiSeq tag sequencing will yield approximately 50,000 reads/sample, ensuring detection of both dominant (core microbiome) and poorly represented taxa (variable microbiome). The hypervariable V3–V5 region was selectively amplified from total genomic DNA by 30 cycles of PCR using conserved primer sequences 357F (CCTACGGGAGGCAGCAG) and 926R (CCGTCAATTCMTTTRAGT) using protocols established by the HMP^27^. Amplicon pools were quantified using a Qubit fluorimeter, and the average fragment sizes were determined on an Agilent bioanalyzer High Sensitivity DNA LabChip (Agilent Technologies, Wilmington, DE) and diluted to 10nM. Amplicons were spiked with 20% of PhiX control library to provide for accurate calculation of matrix, phasing and prephasing. The mixture was sequenced on an Illumina MiSeq V2 sequencer for 250nt from each end of the amplified fragments.

Key OTUs mediating UCPPS were identified using an unbiased protocol that incorporated machine learning and decision tree algorithms (see also below). Data from 16S sequence reads were binned at 97% sequence identity using QIIME and Galaxy to define OTU abundance profiles and phylogenic relationships^50^,^51^. Metagenome analyses were performed using MG-RAST version 3.6 at the Argonne National Laboratory^52^.

### Extended Random Forest

Extended Random Forest was applied to identify and rank OTUs predictive of pain phenotype. This process uses the Random Forest (RF) algorithm followed by stability analysis and feature permutation to determine all relevant OTUs. RF has been used on similar problems in other fields^53^. RF shows excellent performance with this type of complex data and is well suited for datasets where the number of variables is orders of magnitude larger than the number of patient samples^28^,^54^.

For this study of IC/BPS, we first pre-processed the data by dropping features with zero variance, and then we incorporated a parameter-space mapping algorithm to determine the model parameters for RF. Using these parameters (Ntree: 25000, Mtry: 200), RF constructed an ensemble of decision trees and provided VIM scores for each variable. VIM represents the benefit of having a feature included in a tree compared to random noise. For feature ranking, the advantage of the RF permutation-accuracy measure compared to univariate filtering measures is that it captures the impact of each feature individually, as well as conditional importance stemming from multivariate interactions between features^55^.

These steps were followed by stability analysis and feature permutation to identify a minimal optimal set of highly relevant OTUs. The original feature set was extended with a permuted set of features, called shadow features. For each original feature, a shadow feature was created by randomly permuting the values from all observations. When permuted features are used for prediction, the prediction accuracy decreases substantially. Thus a reasonable measure for feature relevance is the likelihood that a feature has a higher mean VIM than its shadow feature. This was estimated by the p-value of a one-sided t-test between each feature and the shadow with the highest mean VIM.

### DIPP species quantitation

Relative levels of species in controls and IC patients were measured by quantitative real-time PCR. DNA was purified from stool samples were using the Qiagen Stool DNA purification kit according to the manufacturer’s instructions. Primers were designed against unique regions of 16S rRNA. The following species-specific primers were confirmed by melt-curve analyses: *O. splanchnicus* forward TCGAAGGCTTGACCTTACGC and reverse TTCATTCGTACGTCCGGTGG; *F. prausnitzii* forward GAGGTTGAACGGCCACATTG and reverse ACCTAGTAAACATCGGCC AC; *E. sinensis* forward GGGATCTCTAATCCGAGGGC and reverse TAATGCGTTAGCTGCGGGC; *E. lenta* forward GGAATGCGCAGATATCGGGA and reverse CCCCCACACCTAGTATCCATC; *C. aerofaciens* forward GTGGAACACCGATGGCGAA and reverse CCTGGTAAGGTTCTTCGCGT; *L. pectinoschiz* forward AGCAGTTGGAAACGGCTGAT and reverse TTACTGACCGGGCAAGGA G; *L. longoviformis* forward TGCATTGGAAACTGTGCAGC and reverse TTCTTGCGAACGTACTCCCC; and *R. intestinalis* forward CGGCTTAAATACGTGCCAGC and reverse AGCCTCAGCGTCAGTAATCG. Duplicate amplification reactions were carried out in a volume of 20 μL that included 10 μL of SYBR Green (BioRad), 0.5 nM stool DNA, and 0.5 nM each primer using the following cycling conditions: 95° for 5 minutes, 40 cycles of denaturing 20″ at 95°, annealing 30″ at 70°, and extension 30″ 72°. Differences in threshold cycles between the positive control and each sample were quantified by the ΔΔCT method and expressed as fold change. ROC estimates were constructed from 5-fold cross validation using a linear threshold model.

### Metabolomics

Two 1ml fractions of the lavage samples were taken from each subject and dried. One fraction was derivatized according to Roessner and colleagues^30^ with minor modifications: 90 minutes at 500 °C with 80 μl of methoxyamine hydrochloride in pyridine (20 mg/ml) followed by a 60 minute treatment at 500 °C with 80 μl MSTFA. A 5 μl aliquot of an internal standard (C31 fatty acid) was added to each, in the derivatized samples this occurred prior to trimethylsilylation. Sample volumes of 1 mL were injected with a split ratio of 7:1 into a GC-MS system consisting of an Agilent 7890A (Agilent Inc, Palo Alto) gas chromatograph, an Agilent 5975C mass selective detector and Agilent 7683B autosampler. Gas chromatography was performed on 60 m HP-5MS columns with 0.25 mm inner diameter and 0.25 mm film thickness (Agilent Inc, Palo Alto) and an injection temperature of 2500 °C, the interface set to 2500 °C, and the ion source adjusted to 2300 °C. The helium carrier gas was set at a constant flow rate of 1.5 ml min^−1^. The temperature program of 5-min isothermal heating at 700 °C, followed by an oven temperature increase of 50 °C min^−1^ to 3100 °C and a final 20 min at 3100 °C. The mass spectrometer was operated in positive electron impact mode (EI) at 69.9 eV ionization energy in m/z 30–800 scan range. The spectra of all chromatogram peaks were then compared with electron impact mass spectrum libraries NIST08 (NIST, Gaithersburg), WILEY08 (Palisade Corporation), and to a custom library of the University of Illinois metabolomics center. To allow direct comparisons between samples all data was normalized to the internal standard in each chromatogram. The chromatograms and smass spectra were evaluated using the MSD ChemStation (Agilent, Palo Alto) and AMDIS (NIST, Gaithersburg). The retention time and mass spectra were implemented within the AMDIS method formats.

Metabolomic data were analyzed using Pathway Analysis and ROC Analysis modules in MetaboAnalyst 3.0^31^,^32^,^33^. Pathway analysis utilized normalization to sample mass for analytes in the Human Metabolome Database ID. ROC analyses were performed by univariate analysis.

### Predictive functional analysis

Phylogenetic Investigation of Communities by Reconstruction of Unobserved States (PICRUSt) was used to predict function from 16S sequence data, PICRUSt predicts function from phylogeny based on the shared gene content and does not consider the variations among species^56^. The PICRUSt compatible OTU table was further normalized and used to predict to KEGG Orthologs (KOs) using the online PICRUSt Galaxy version.

## Additional Information

**How to cite this article**: Braundmeier-Fleming, A. *et al.* Stool-based biomarkers of interstitial cystitis/bladder pain syndrome. *Sci. Rep.* **6**, 26083; doi: 10.1038/srep26083 (2016).

## Figures and Tables

**Table 1:** Study participants.

	Healthy controls (n = 16)	IC patients (n = 18)	Significance
Age (yrs)	35 ± 11	35 ± 9	
Sex	Female	Female	
Race	6 African-American 10 Caucasian	0 African-American 18 Caucasian	
Employment	0 disability	3 disability	
Family history of UCPPS	0	2	
Genitourinary urinary pain index (GUPI) score, total	1 ± 2	26 ± 8	p < 0.0001
GUPI, pain subscore	N/A	13 ± 4	p < 0.0001
GUPI, urinary symptoms subscore	1 ± 1	4 ± 3	p < 0.0001
GUPI, QoL subscore	N/A	8 ± 3	p < 0.0001
Symptoms duration (yrs)	N/A	9 ± 8	
Genitourinary disorder	2	11	
Allergies, respiratory tract disorder	4	14	
Gastrointestinal disease	0	6	
Neurologic disease	0	5	
Urologic/gynecologic surgeries	1	7	
Currently receiving treatment	0	13	

**Table 2:** Stool-based IC biomarkers.

DIPP Species	ROC area under curve (mean)
*Colinsella aerofaciens*	0.86
*Eggerthella sinensis*	0.84
*Faecalibacterium prasunitzii*	0.79
*Odoribacter splanchnicus*	0.72
*Lactonifactor longoviformis*	0.55
Metabolites	
Glyceraldehyde	0.98
